# MYCN Function in Neuroblastoma Development

**DOI:** 10.3389/fonc.2020.624079

**Published:** 2021-01-27

**Authors:** Jörg Otte, Cecilia Dyberg, Adena Pepich, John Inge Johnsen

**Affiliations:** Childhood Cancer Research Unit, Department of Children’s and Women’s Health, Karolinska Institutet, Stockholm, Sweden

**Keywords:** MYCN, neuroblastoma, childhood cancer, neural crest, cancer stem cell

## Abstract

Dysregulated expression of the transcription factor MYCN is frequently detected in nervous system tumors such as childhood neuroblastoma. Here, gene amplification of *MYCN* is a single oncogenic driver inducing neoplastic transformation in neural crest-derived cells. This abnormal *MYCN* expression is one of the strongest predictors of poor prognosis. It is present at diagnosis and is never acquired during later tumorigenesis of *MYCN* non-amplified neuroblastoma. This suggests that increased *MYCN* expression is an early event in these cancers leading to a peculiar dysregulation of cells that results in embryonal or cancer stem-like qualities, such as increased self-renewal, apoptotic resistance, and metabolic flexibility.

## Introduction

*MYCN* belongs to a small family of genes, which in addition to *MYCN* (or *N-Myc*) includes two closely related genes, *C-Myc* and *L-Myc*. MYC proteins are master regulators of cell fate and part of a network of interacting transcription factors. Together, these transcription factors regulate the expression of multiple genes involved in cell-proliferation, growth, senescence, metabolism, differentiation, and apoptosis (1). More specifically, MYC proteins bind to active promoters and enhancers altering transcription mediated by all three RNA polymerases and affecting the expression of more than 15% of all genes in a cell (2–4). Dysregulated expression of *MYC* genes are frequently observed in cancers of different origin, implicating that MYC proteins have central functions during carcinogenesis (2–4). Additionally, MYC proteins also affect the tumor microenvironment; MYC protein was shown to regulate the interaction between tumor cells and the host immune cells by controlling the synthesis of cytokines mediating communication between tumor cells and myeloid cells (5–7).

Studies in mice showed that both *Myc* (*C-myc)* and *Mycn* (*N-myc)*, but not *Mycl* (*L-myc*), are fundamental for normal development as targeted deletions of these two genes in mice are embryonic lethal at mid-gestation (8–12). While *C-myc* is expressed throughout the mouse embryo and at all developmental stages analyzed; *N-myc* expression is restricted to hematopoietic stem cells and cells within the developing nervous system (12–16). The restricted expression pattern of *N-myc* during development may be mirrored in human tumors since cancers with a neural cell origin like neuroblastoma, medulloblastoma, retinoblastoma, astrocytoma and glioblastoma, as well as, hematological malignancies frequently overexpress *MYCN*. However, overexpression of *MYCN* has also been reported in Wilms tumors, rhabdomyosarcomas, prostate, pancreatic and lung cancers (17). The mechanisms for *MYCN* overexpression in tumors have several facets, ranging from induced transcriptional activation of *MYCN*, increased MYCN protein stabilization caused by dysregulated MYCN phosphorylation, and reduced proteasomal degradation to *MYCN* gene amplification (17–19). Pediatric cancers usually develop during a much shorter time-period and with significantly fewer genetic abnormalities compared to adult tumors. Some of these childhood tumors have embryonal characteristics that are most probably initiated by aberrations in genes and/or deregulated expression of genes causing retention of cell immaturity and increased proliferation capacity (18). In mice models, guided ectopic expression of N-myc to the developing nervous system has been shown to be a potent oncogenic driver and results in the development of medulloblastoma and neuroblastoma (20–22).

## MYCN in Neuroblastoma

Neuroblastoma is a cancer of the peripheral nervous system that almost exclusively occurs during early childhood. Although neuroblastoma is a relatively rare disease affecting 1 of 8,000 live births, or 6%–10% of all childhood tumors, the disease still accounts for 12%–15% of all cancer-related deaths in children. 40% of the patients diagnosed with neuroblastoma are younger than 1 year and the median age for diagnosis is 17-18 months whereas less than 5% of the patients are older than 10 years establishing neuroblastoma as the most common and deadly tumor of infancy (23). Clinically, neuroblastoma is characterized with a heterogeneous disease spectrum ranging from patients with widespread tumors that spontaneously regress or differentiate without treatment to treatment-resistant tumors with metastatic spread despite intensive multimodal treatment approaches. This heterogeneity is mirrored in overall patient survival; neuroblastoma patients with low- to intermediate-risk disease have 85-90% survival rates, whereas 50% of patients with high-risk neuroblastoma succumb to the disease.

The risk stratification of low-, intermediate- and high-risk patients usually becomes evident through chromosomal analysis. Low-risk neuroblastoma commonly displays whole chromosomal gains with a hyperdiploid (near triploid or penta/hexaploid) chromosomal landscape, whereas high-risk neuroblastoma contains segmental chromosomal aberrations that affect only a part of a given chromosome. The most common chromosomal aberration related to poor prognosis in neuroblastoma is somatically acquired segmental gain of 17q, hemizygous deletions of 1p and 11q, and *MYCN* gene amplification. In addition, genomic rearrangements at chromosomal region 5p15.33, located proximal of the telomerase reverse transcriptase gene (*TERT*) that results in chromosomal changes, DNA methylation and enhanced TERT expression have also been observed in high-risk neuroblastoma samples. Gene amplification of *MYCN* was one of the earliest genetic markers discovered in neuroblastoma and is still one of the strongest predictors of poor prognosis. The prevalence of *MYCN* amplification in neuroblastoma patients is 20%–30% and the overall survival for these patients remains at less than 50% (23–25). In high-risk neuroblastoma patients, if amplification of *MYCN* occurs it is always present at diagnosis. Patients with low-risk disease lack *MYCN* gene amplification and never progress to high-risk disease nor do they acquire extra copies of the *MYCN* gene (17, 23, 26). This indicates that *MYCN* gene amplification is an early and perhaps initiating event driving the development of a high-risk neuroblastoma subgroup of tumors which is in contrast to most other cancers where gene amplifications are considered to be late events during tumorigenesis (17, 27).

Adult cancer is a multistep process that evolves over many years caused by genomic instabilities giving rise to transformed cells that have the capacity to develop into life-threatening malignant tumor cells. Pediatric cancers, on the other hand, develop during a short time-period and contain much fewer genomic aberrations and mutations compared to adult cancers. This suggests that certain pediatric cancers, including neuroblastoma, arise from cells with embryonal features or from mature prenatal cells that through external factors have acquired embryonal properties favoring proliferation (18). Although *MYCN* gene amplification is detected in approximately 50% of high-risk neuroblastoma cases and an oncogenic driver for neuroblastoma, there exists no current evidence describing when and how the amplification of the *MYCN* gene is initiated. Neither is it known in detail how and in which cell type the expression of *MYCN* is initiated in order to drive neuroblastoma tumorigenesis. Furthermore, it should be noted that the 2p24 chromosomal amplicon observed in high-risk neuroblastoma encodes other genes in addition to *MYCN*, such as the anaplastic lymphoma kinase (*ALK)*. *ALK* has also been shown to drive neuroblastoma formation and to potentiate the oncogenic activity of *MYCN* in neuroblastoma (28–30).

## Neuroblastoma Is a Neural Crest Derived Malignancy

**Figure 1:**
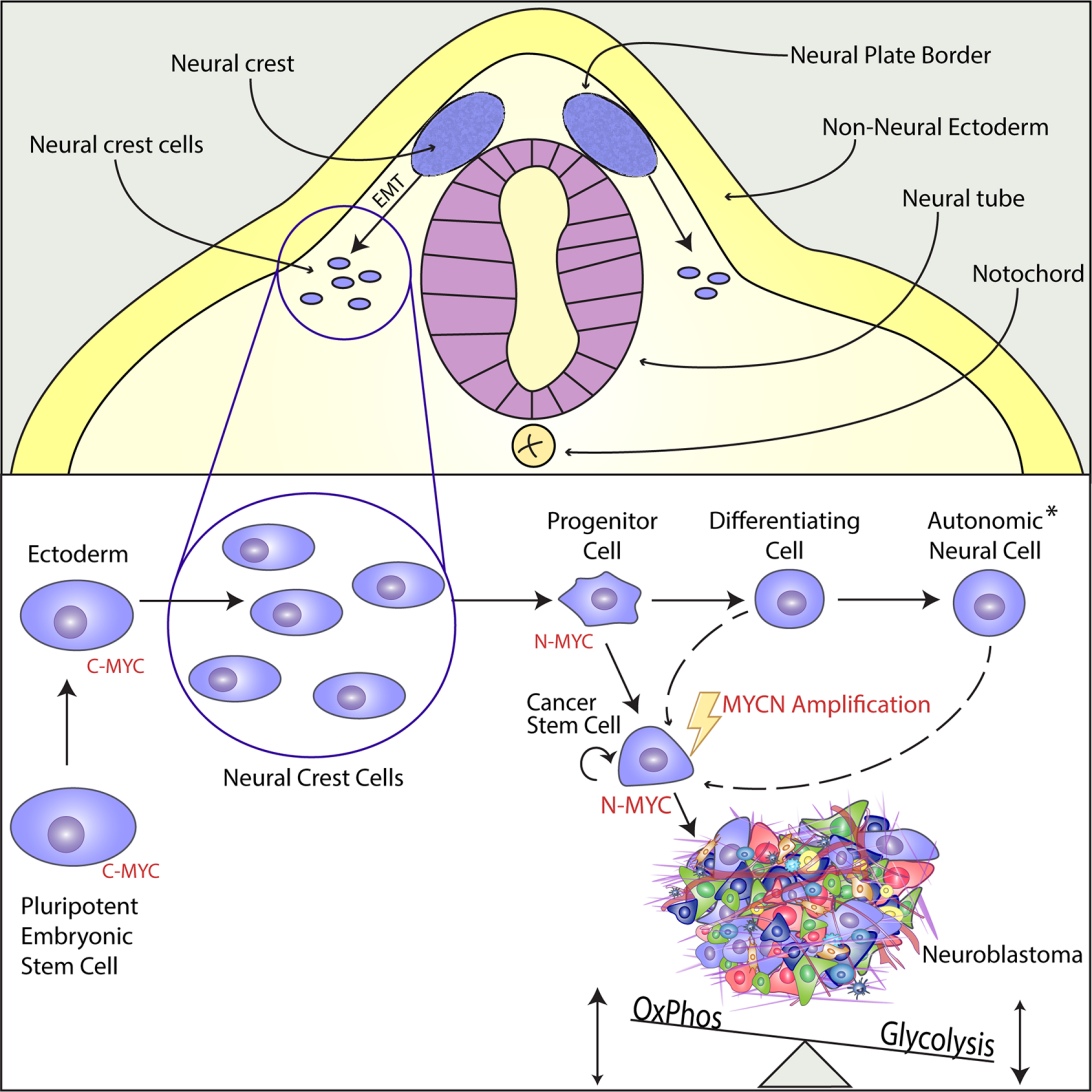
N-MYC acts as a Cancer Stem Cell Factor in the Developing Neural Crest and Promotes Tumorigenesis in Neuroblastoma. Upper Panel: The neural crest is a transient structure located in the neural plate border, an area between the neural plate and the non-neural ectoderm. From the neural crest, multipotent progenitor cells delaminate, migrate through epithelial-to-mesenchymal-transition (EMT), and differentiate into versatile structures within the whole organism. Lower Panel: While C-Myc is the main regulator in pluripotent cells of early embryonal development, MycN is highly expressed in the multipotent cells of the migratory and post-migratory neural crest. During differentiation, MycN expression is downregulated and the sympathoadrenal precursor cells or progenitor cells mature into different cell types of the autonomic neural cell lineage (see asterisk), such as sympathetic ganglion cells, chromaffin cells of the adrenal medulla or cells of the peripheral nervous system. Even though there is strong evidence that *MYCN* gene amplification is an early and maybe initiating event, it has not been proven yet when and how the amplification takes place. The aberrant expression of MYCN induces a unique cancer stem cell-like phenotype by enabling infinite self-renewal, apoptotic resistance, and *via* metabolic reprogramming characterized by increased glycolysis together with an active oxidative phosphorylation. The establishing neuroblastoma consists of heterogeneous cell populations.

Neuroblastoma derives from cells within the neural crest, a transient structure consisting of multipotent progenitor cells present during embryogenesis. The majority of the tumors are located in the abdomen along the sympathetic chain and in the adrenal gland medullary region (23, 27). The neural crest develops between the neural plate and non-neural ectoderm, in an area named the neural plate border, during gastrulation and neurulation (**Figure 1**) (31). Neural crest cells undergo epithelial-to-mesenchymal transition (EMT) during neurulation and migrate extensively from the neuroepithelium to more distant locations in the embryo where the cells differentiate into a wide variety of cell types for specific organ systems including the peripheral and enteric nervous systems, skin pigment, cardiovascular system and craniofacial skeleton (32). Neural crest cell maturation, migration, specification and differentiation are tightly controlled processes guided by gene regulatory networks, consisting of various transcription factors. These transcription factors become sequentially activated by external factors like BMPs, Wnt and FGF (33). The cell of origin for neuroblastoma has yet to be determined, but the combination of timing in disease onset and clinical presentation suggest that neuroblastoma is derived from sympathoadrenal progenitor cells within the neural crest that differentiate to sympathetic ganglion cells and adrenal catecholamine-secreting chromaffin cells.

Recent reports, based primarily on *in vitro* studies of neuroblastoma cell lines, have demonstrated that neuroblastoma consists of two phenotypically different subpopulations of cells, called adrenergic and mesenchymal. These two subpopulations show distinct networks of super enhancer-associated transcription factors (34, 35). *In vitro*, these cell lineages can interconvert and exhibit differences in sensitivity to chemotherapeutic drugs. Interestingly, the mesenchymal population of cells seems to be more drug resistant which may be important for the development of drug resistance frequently observed in patients with high-risk neuroblastoma (19, 34). The identity of a cell from the adrenergic subtype is defined by key regulatory genes, such as *PHOX2B*, *HAND2*, or *GATA3*, which are interconnected by reciprocal regulation (35). This core regulatory circuitry can be reinforced by MYCN amplification through a mechanism called “enhancer invasion”. Enhancer invasion depends on combined enhancer co-occupation of individual gene enhancer/promoter regions by MYCN and TWIST1 (36). Another regulatory loop involving MYCN has recently been identified and depends on ASCL1 which is directly regulated by MYCN together with LMO1. In the same study, it has been shown that *ASCL1* is, itself, a part of the core regulatory circuitry under the adrenergic identity and expression of *ASCL1* can induce differentiation arrest in neuroblastoma cells (37).

Overexpression of *Mycn* in migrating neural crest cells of chicken embryos increases the proportion of neurons at the cost of other cells. Loss of *Mycn* in mouse embryos decreases the size of the entire nervous system, including spinal, peripheral and cranial ganglia and reduces the number of mature neurons in the spinal ganglia (9). In the sympathetic ganglia, expression of *C-myc* is considerably higher compared to *N-myc*. This indicates that *N-myc* expression is induced in the sympathetic ganglia during gestation and then switched off before birth (38). Interestingly, the expression level of *C-myc* does not change after *N-myc* downregulation. On the other hand, downregulation of *N-myc* strongly upregulates the expression of *Phox2b*, *Mash1, Hand2*, and *Gata3* genes.

## *MYCN* as an Oncogenic Driver in Neuroblastoma

Despite that several molecular prognostic factors with oncogenic potentials have been described in neuroblastoma, only activating *ALK* mutations and *MYCN* overexpression were shown to be *de novo* oncogenic drivers. This is seen when mutation or overexpression of these molecules give rise to neuroblastoma in genetically engineered animal models.

The *ALK* gene located on chromosome 2p23 encodes a receptor tyrosine kinase that is normally expressed at high levels in the nervous system and was originally identified as a fusion protein in non-Hodgkin’s lymphoma. Zhu et al. generated transgenic zebrafish models in which human *MYCN*, human *ALK* or *ALK* harboring the F1174L activating mutation (*ALK^F1174L^*) were placed under control of the dopamine β-hydroxylase (*dbh*) promoter (30). F1174L mutation is one of the most frequent activating somatic mutations in human cell lines and neuroblastoma patients (39). Tumor penetrance and the rate of tumor induction were much higher in zebrafish expressing both *MYCN* and *ALK* compared to zebrafish expressing either *MYCN* or *ALK*. Additionally, zebrafish expressing both *MYCN* and *ALK^F1174L^* transgenes demonstrated increased tumor penetrance compared to all other transgene combinations. The expression of *alk* increased the effect of *mycn* by blocking apoptosis in *MYCN*-overexpressing sympathoadrenal neuroblasts (30). *ALK* mutations can be detected in all clinical stages while an association with a poorer outcome can only be detected in intermediate- and high-risk neuroblastoma. In 2%–3% of all cases, *ALK* can be further activated by gene amplification leading to protein expression and kinase activity. Interestingly, this is specific to neuroblastoma as it has been described that *ALK* amplification does not result in protein expression in non-small-cell lung cancer and is therefore not involved in its pathogenesis but maybe only a passenger event (40–42).

Targeted expression of *Lin28b* to sympathetic adrenergic lineage cells gives rise to neuroblastoma. One mechanism of induced tumor development indicates a Lin28B-mediated downregulation of Let-7 which results in high MYCN protein expression suggesting that *MYCN* is the actual driver of oncogenesis (43). Others have described let-7-independent pro-tumorigenic effects of LIN28B e.g. *via* protein-protein interaction with the transcription factor ZNF143 recruiting LIN28B to activate promoters of genes involved in neuroblastoma progression (44, 45).

*MYCN* is highly expressed in the early post-migratory neural crest (**Figure 1**) and regulates ventral migration and growth of cells within the neural crest during normal murine sympathoadrenal development. *MYCN* expression is gradually downregulated in differentiating sympathetic neurons, which suggest that sympathoadrenal maturation is independent of *MYCN* expression. The sympathoadrenal precursor cells maturate into neural or chromaffin cells. Moreover, it has been proposed that the preneoplastic lesions, which can develop into neuroblastoma, arise in sympathoadrenal precursor cells not having received or reacted to signals controlling the neuronal or chromaffin cell fate. Studies in zebrafish demonstrated that ectopic expression of *mycn* in sympathoadrenal precursor cells obstruct the development of chromaffin cells causing the development of neuroblastoma. An excess of precursor cells is produced during the transition to sympathoadrenal cells, which during normal stages of maturation, are submitted to controlled apoptosis caused by deficiency of local neural growth factors. As MYCN is a master transcription factor important for both proper cell proliferation and apoptosis, a persistent expression of MYCN during the maturation stages of sympathoadrenal precursors could result in inhibition of apoptotic signaling and maintained proliferation that ultimately could result in the development of neuroblastoma (17).

In a genetically engineered neuroblastoma mouse model, called Th-MYCN (22), accumulation of small, blue round cell populations in the paravertebral ganglia are observed at embryonic day 14 (46). This population of cells called neuroblasts later develops into neuroblastoma observed in 100% of the Mycn homozygous mice from postnatal week 6. Similar observations were shown in transgenic zebrafish with neural crest cells expressing mycn. Furthermore, overexpression of *MYCN* in primary neural crest cells isolated from an embryonic neural tube explant developed tumors that were highly similar to *MYCN* amplified neuroblastoma when the cells were inoculated in mice (47). However, blocking expression of *Mycn* in neural crest cells was recently shown to induce perinatal lethality in mice which suggests that primary neural crest cells are not the origin of *MYCN* amplified neuroblastoma (48). This is in line with a study showing that *Mycn* is expressed together with the phosphorylating-stabilizing factor, *CIP2A* in regions of the neural plate and that Mycn protein is excluded from the neural crest stem cell domain (49). This indicates that high-risk subgroups of neuroblastoma may be initiated before the emigration of neural crest cells and before sympathoadrenal specification. The importance of *MYC* expression in neuroblastoma is further emphasized by the fact that neuroblastoma without *MYCN* gene amplification frequently expressed high levels of *C-MYC* indicating that *C-MYC* and *N-MYC* are mutually exclusive in neuroblastoma (50).

## MYCN as a Stem Cell Factor

The interrelation of neural crest development and neuroblastoma tumorigenesis is one good example of how developmental biology and cancer research fuel each other in their mutual discoveries. In fact, it was the early analysis of teratocarcinomas that led to the discovery of embryonic stem cells (ESC) (51). The theory of cancer stem cells has elucidated the exploitation of embryonic pathways by malignant cells, not only in pediatric tumors, but in most cancers. More recent studies have further demonstrated that malignant as well as non-malignant tissues can possess unexpected plasticity (52, 53).

The Nobel-prize awarded discovery of induced pluripotency by Yamanaka in 2006 has opened a new chapter in regenerative medicine and demonstrated a paradigm shift in our understanding of cellular plasticity and lineage restriction (54). By reprogramming fibroblasts to become induced pluripotent stem cells (iPS) with activation of merely four transcription factors (*SOX2*, *OCT4*, *KLF4*, and *C-MYC*), Yamanaka showed that no definite cell state is truly irreversible. *KLF4* and *C-MYC* are well known oncogenes, indicating similarities in reprogramming and tumorigenesis. The oncogenic potential of iPS-derived cells is a major concern in today`s regenerative medicine (55) and has led to a deeper analysis of *C-MYC* and its tumorigenic potential during the process of reprogramming. Other protocols for the generation of iPS cells without direct *MYC* activation have been described, although they usually have a lower reprogramming efficiency (56).

One important hallmark of all stem cells is their continuous self-renewal which can be described as proliferation without differentiation. By inhibiting the expression of cell lineage specifiers while simultaneously inducing cell cycle progression, MYC proteins fulfill a major function in establishing self-renewal in ESCs, as well as in cancer stem cells (1, 57, 58).

During early embryogenesis in mice, C-*myc* and N-*myc* are highly redundant and can compensate each other’s loss. Single knock-outs have no effect on the self-renewal or pluripotency of mouse ESCs, while N-*myc* can be substituted for C-*myc* during reprogramming (10, 57, 59, 60). However, later during development *Myc* expression becomes tissue specific and single knock-outs of *C-myc* and *N-myc* become lethal during mid-gestation (10, 12, 16, 26). Besides tissue specific expression, Myc proteins are highly conserved in their structure and function which has been proven in murine development by transgenic mice expressing *N-myc* from the *C-myc* gene locus which generated healthy and fertile offspring (61). These along with other studies have shown that C-myc and N-myc fulfill similar functions as stem cell factors, but tissue specific differences have been described within their networks of protein interactions and transcriptional regulation (62). Insights obtained mainly by the analysis of C-myc as a pluripotency factor can, to a certain degree, be transferred to N-myc’s role in stem cell research and during oncogenesis.

## MYCN as an Apoptosis Regulator

Oncogenic transformation and cellular reprogramming are similar processes that are impeded by cell intrinsic barriers. A critical mechanism is the induction of apoptosis or senescence facilitated by p53, the most commonly mutated gene in the Pan-Cancer cohort of The Cancer Genome Atlas (TCGA) (63). Balance in p53 activity is essential for self-renewal of undifferentiated cells and is tightly regulated at the mRNA-, as well as at the protein-level (64). During reprogramming, p53 acts as a roadblock. Thereby p53 inhibition increases reprogramming efficiency, but at the cost of increased oxidative stress, shortened telomeres and higher risk of DNA damage (65). Results by Olsen et al. have indicated that cultured primary mouse neural crest cells with a heterozygous p53 deletion (p53^+/-^) allow more permissive tumor induction by lentiviral *MYCN* transduction than p53 wild type cells (47). In the vast majority of human neuroblastoma, however, no p53 mutations are detectable at diagnosis and tumor cells express nuclear protein as well as a functional cytochrome c-caspase cascade (66–68). C-myc and N-myc can both bind the p53 promoter and induce its expression without eliciting apoptosis indicating additional anti-apoptotic effects regulated by Myc (69, 70). Many players in this complex network have been studied extensively constituting promising candidates for future targeted therapies (71). The main antagonist of p53 is Mdm2, an E3–ligase contributing to the ubiquitination and the repression of p53. Mdm2 is directly induced by N-myc but also regulates N-myc in a feedback-manner (72, 73). In neuroblastoma, Mdm2 can acts as a tumor promoting factor as seen in Mdm2 haploinsufficient (*Mdm2^+/-^)* MYCN transgenic mice which show a decreased tumor incidence, latency, and reduced tumor growth (74). The same study described that *Mdm2^+/-^* tumors had a decreased level of p19^Arf^ (Cdkn2a), another tumor suppressor that is reciprocally regulated by N-myc. p19^Arf^ in turn can be inhibited by Twist-1 which is also a direct target of N-myc (75). The upregulation of Twist-1 correlates with *MYCN*-amplification in neuroblastoma indicating that apoptosis evading mechanisms are different in *MYCN* amplified compared to non-amplified neuroblastoma (76). A direct targeting of the *MYCN* gene using siRNA, CRISPR/Cas9 crRNA molecules or specific alkylating agents induced apoptosis in *MYCN* amplified cells but not in non-amplified cells (77). These studies indicate that N-myc alone can prevent apoptosis or senescence in neuroblastoma without depending on mutations in additional tumor-promoting genes. N-myc regulates the transcriptional network around p53 to overcome these intrinsic barriers, similar to the artificial activation of the pluripotency program in healthy somatic cells. Why the incidence of initial p53 mutations in neuroblastoma is only around 2% remains an open question, especially if the selection pressure during early tumorigenesis preferentially selects cells with an active p53 signaling (67). In early analysis of heterozygous p53-deficient mice (*tp53^+/-^*), it was observed that a high proportion of tumors retain the functional copy of p53 while only a minor fraction lost their wild-type allele. The functional copy of p53 prevented chromosomal instability and induced apoptosis after radiation therapy (78).

During the early onset of neuroblastoma, the cell of origin depends on self-renewal to maintain tumor growth as a homeostatic process. It is known from pluripotent cells that increased cellular stress can disturb this homeostasis, inhibit self-renewal and induce differentiation or senescence. P53 can alleviate cellular stress by DNA damage control or by reducing oxidative phosphorylation (65). Downstream effects of MYCN might induce a transient reduction of p53 protein, e.g. during G1 cell cycle checkpoint phase. There is further evidence that micro RNAs such as miR-380-5p contribute to this fine-tuned balance (79). Chemotherapy, however, disturbs this balanced activity of p53 by inducing massive DNA damage forcing p53 wild-type tumors to regress. Many high-risk neuroblastomas relapse as a therapy-resistant metastatic disease with increased frequency of mutations in P53-MDM2- p19^Arf^ pathway (80).

## MYCN Confers Metabolic Plasticity

During embryonic development, stem cells undergo rapid cell duplications while passing through different cell states in a continuously changing environment. Their metabolism is challenged to provide a sufficient amount of energy, in the form of ATP, but also to generate molecules for biosynthetic demands such as DNA replication or cell growth. This versatile flexibility is re-acquired by somatic cells during reprogramming and is also shared by cancer cells (81).

Differentiated somatic cells usually generate energy by metabolizing glucose to carbon dioxide. In the initial anaerobic glycolytic step, glucose is oxidized to pyruvate which can be converted into lactate. Alternatively, pyruvate can enter the mitochondrial matrix where it is further catabolized to Acetyl-CoA feeding the tricarboxylic acid (TCA) cycle. The TCA cycle takes part in cellular redox reactions by providing reduced oxidizing agents such as NADH and FADH as electron sources for oxidative phosphorylation (OxPhos), which is the mitochondrial direct ATP synthesis pathway when under an aerobic state. While OxPhos produces vastly more ATP per glucose molecule, ATP generation is faster during anaerobic glycolysis (82, 83). The observation that cancer cells fulfill a glycolytic switch, meaning they depend mostly on anaerobic glycolysis despite a sufficient oxygen supply, is referred to as the Warburg effect or aerobic glycolysis (82).

Otto Warburg stated in his article from1956, “On the Origin of Cancer Cells”, that the “irreversible injury of respiration” constitutes the first phase of cancer development (84). A deeper understanding of the cellular metabolism has revealed that cataplerosis, the use of partially oxidized metabolites from the TCA cycle, is an important and eclectic source for versatile anabolic processes (85). Thus, pyruvate and alpha-ketoglutarate, key intermediates in the TCA cycle, are important building blocks for non-essential amino acids and Acetyl-CoA is the precursor molecule of fatty acid synthesis and histone acetylation. Further, direct derivatives of glucose can be incorporated into the pentose phosphate pathway providing ribose for nucleotide synthesis (82, 85). Otto Warburg couldn’t be aware that the incomplete oxidation of substrates constitutes an advantage for the anabolic processes in cancer tissue despite a less efficient ATP generation. The investigation of embryonic stem cells and later of induced pluripotent stem cells has shown, however, that dependency on glycolysis in pluripotent stem cells and cancer cells is not a consequence, but a pre-requisite of successful embryonal development and tumorigenesis (81).

ESCs can obtain different states of pluripotency, of which the naïve state better reflects the ground state pluripotency of the inner cell mass from the preimplantation blastocyst than the primed pluripotency state. While MYC proteins are crucial in order to maintain pluripotency in both states, human naïve pluripotent cells show a specific nuclear expression of MYCN associated with a higher glycolytic flux (86). If the glycolytic enzyme hexokinase II is pharmacologically inhibited by the pyruvate analog 3-bromopyruvate, cultured ESCs switch from anaerobic glycolysis to OxPhos and lose their pluripotency, even under pluripotency promoting conditions indicating the functional role of the glycolytic metabolism in pluripotency (87). Likewise, the generation of iPS from somatic cells highly depend on functional glycolysis as its inhibition reduces reprogramming efficiency while it is supported by the stimulation of glycolytic activity (88). Several studies have indicated that the metabolic restructuring during reprogramming precedes the expression of pluripotency factors. The expression of MYC in cancer cells or as a transduced factor during reprogramming supports this switch by inducing hypoxia inducible factor 1a (Hif1A) and its downstream targets pyruvate dehydrogenase kinase and other glycolytic enzymes (88–91). However, a study in MYC inducible cancer cell lines has shown that highly proliferating MYC expressing cells require active OxPhos together with increased glycolysis to drive their fast cell cycle progression (92).

Similar results have been described in neuroblastoma, where *MYCN*-amplified cells display a distinct metabolic structure defined by high energy consumption and production compared to *MYCN* non-amplified neuroblastoma cells (93, 94). In a detailed metabolic analysis of *MYCN*-amplified neuroblastoma cells, Oliynyk et al. showed that *MYCN* induction upregulates glycolytic enzymes such as hexokinase-2 but that the dominant effect was an increase in OxPhos induction (94). Interestingly, *MYCN* activation not only increased the oxygen consumption rate but also OxPhos response provoked by metabolic stress. This indicates increased flexibility for regulating glycolysis or mitochondrial respiration within MYCN expressing cells (94).

Beyond glucose, glutamine and fatty acids are important fuels for mitochondria generating ATP and other macro-molecules. MYCN enables neuroblastoma cells to oxidize fatty acids with a higher capacity than in non-*MYCN* amplified cells. This feature might become of clinical relevance as it has been shown that the inhibition of β-oxidation induces differentiation in MYCN expressing tumor spheroids and leads to a decreased tumor growth in *MYCN* amplified cell lines when injected into nude mice (94). Another metabolic hallmark of many cancers is increase in glutaminolysis. Glutamine is a versatile nutrient and its derivatives are involved in many anabolic processes within the cell. Its carbon atoms can be fed into the TCA cycle or can be utilized to generate amino or fatty acids. Glutamine further provides nitrogen for amino acid production as well as for nucleotide biosynthesis (95). Highly proliferating cancer cells are often addicted to glutamine and studies have shown that MYCN leads to an upregulation of glutaminolytic enzymes, while also selectively inducing apoptosis in glutamine depleted cells (96, 97). Although, a recent *in vitro* study found that MYCN expression can enable tumor cells to synthesize glutamine from glucose-derived alpha-ketoglutarate (94, 98). The ability to adapt to a low-glutamine environment is highly beneficial for proliferating cancer cells in a poorly vascularized environment. This has therapeutic implications as the glutamine metabolism is currently studied as an anticancer target (95).

The above-mentioned studies have shown that the metabolism of *MYCN* amplified cells is not only driven by the strong proliferative stimulus but remains flexible to provide high amounts of energy as well as bio-macromolecules for anabolic processes. This metabolic structure of a regulated balance between active OxPhos and glycolysis has also been described during the first days of somatic reprogramming as well as in early naïve ESCs being a prerequisite to successfully achieve pluripotency (89, 99–101). Direct comparisons of *MYCN* amplified versus non amplified cells have shown that MYCN is one of the metabolic master regulators equipping cells with a high versatility resembling mechanisms of self-renewing stem cells.

## Self-Renewal in *MYCN* Non-Amplified High-Risk Neuroblastoma

*MYCN* amplification drives tumorigenesis in neural crest cells by maintaining or re-establishing embryonic features in these cells. By conferring stem-like qualities such as infinite self-renewal, apoptotic resistance or metabolic flexibility, MYCN contributes to the life-threatening characteristics of high-risk neuroblastoma. Even though, *MYCN* gene amplification accounts for 50% of all high-risk neuroblastoma cases, the malignant *MYCN* non-amplified neuroblastomas never gain secondary gene amplifications during tumor progression or relapse, which is an uncommon feature of oncogenes in adult cancers (17). High-risk, *MYCN* single copy neuroblastomas often express MYC as the oncogenic driver while MYCN is not expressed in these cells (50). Usually, only one of these two MYC proteins can be expressed in a tumor cell at a time, with MYC often dominating over MYCN by repressing *MYCN* expression (50, 102). Coincidentally, higher MYCN expression in *MYCN* non-amplified tumors was reported to be correlated with favorable prognosis (50, 103). It must be mentioned that these tumor cells never reach the MYCN mRNA expression level of *MYCN* amplified tumors. Most MYC target genes are regulated in a dose dependent manner and non-*MYCN*-amplified tumor cells that artificially overexpress MYCN retain their ability of neuronal differentiation (104, 105).

Inducing stemness without direct MYC overexpression has been shown by reprogramming somatic cells without using MYC. Interestingly, similar pathways that are used to avoid MYC transduction in iPS generation are potentially involved in the tumorigenesis of *MYCN* non-amplified neuroblastomas.

Lin28b, as one example, has been a part of the pluripotency factors first used in reprogramming of human somatic cells that did not depend on the enforced overexpression of MYC (106). It has always been suspected, however, that Lin28b expression leads to an indirect activation of endogenous MYC proteins (56).

Another strategy involves the activation of the Wnt pathway, a well described and highly conserved signaling cascade in embryonal development, tissue stem cells and tumorigenesis. Together with MYC proteins and other factors, Wnt signaling is involved in the initial induction of neural crest cells, their maintenance and later cell fate determination (33, 107). High-risk *MYCN* non-amplified neuroblastoma often have an increased Wnt activity which contributes to its high aggressiveness by inducing *c-MYC* expression (108). A reciprocal mechanism has been described for the Wnt-inhibitor *Dickkopf-3* (DKK3) which induces tumor cell maturation and correlates with a favorable prognosis. In *MYCN* amplified tumors, Dkk3 is often downregulated by MYCN leading to an undifferentiated phenotype and higher aggressiveness (109). With the help of strong Wnt signaling, artificial *c-MYC* activation becomes dispensable when self-renewal and pluripotency is re-induced in differentiated somatic cells (110). Of the aforementioned mechanisms, activation of endogenous *MYC* genes is most closely associated with high risk in *MYCN* non-amplified neuroblastoma. Comprehensive transcriptome analyses elucidates the expression of MYC target genes and their embedded pathways, which can be indicative for the clinical outcome, independent of *MYCN* amplification (111). If the expression of MYC downstream factors such as Hif or the Krüppel-like family of transcription factors (Klf) is highly expressed, the oncogenic activity of MYC becomes irrelevant (112).

## Preclinical *In Vivo* Models of Neuroblastoma

Weiss et al. have previously demonstrated that Mycn has the potential to drive neuroblastoma in a transgenic model mouse model, i.e. *Th-MYCN* mice, which carry, in their germline, human *MYCN* cDNA under the control of the rat tyrosine- hydroxylase promoter (22). Neuroblastoma growth in these *Th-MYCN* mice begins with hyperplastic lesions in sympathetic ganglia through the first few weeks after birth (58). Mice containing *MYCN* transgene targeted to the neural crest cells develop neuroblastoma with a phenotype very similar to the human neuroblastoma (46).

Incorrect MycN expression shortly after birth in the paravertebral ganglia caused neuroblast hyperplasia in *Th-MYCN* mice. *N-myc* amplification existed at low levels in perinatal neuroblast hyperplasia from both hemizygote and homozygote mice. The level of N-myc in hyperplasias and tumor tissue was highest at week 1 of age. A stepwise increase of *N-myc* amplification was only seen in tumor formation of hemizygote mice. The neuroblast hyperplasia in the ganglia from *Th-MYCN* did not express differentiation markers, such as beta-III-tubulin or tyrosine hydroxylase, differing from nearby neuronal cells (46).

Althoff et al. generated a transgenic mice, termed *LSL-MYCN;Dbh-iCre*, with Cre-conditional induction of *MYCN* in *Dbh*-expressing cells. These mice form tumors irrespective of strain background with an incident of 75% (113).

In the *Th-MYCN* mouse model, tumor penetrance is only high in a 129 x 1/SvJ strain background. Tumors in *LSL-MYCN;Dbh-iCre* mice arise in superior cervical ganglion, celiac ganglion or the adrenals covering all locations, in which human neuroblastomas arise. They consist of small blue round cells harboring neurosecretory vesicles. The cells express neuroblastoma specific genes such as paired-Phox2b, Dbh, Th, and high levels of N-myc compared to normal tissue. The level of differentiation and tumor location resemble the human neuroblastoma more in the *LSL-MYCN;Dbh-iCRe* than in the Th-MYCN model.

Hierarchical clustering shows that tumors from the *Th-MYCN* mouse model and *LSL-MYCN;Dbh-iCre* mice are very similar, both at miRNA and mRNA level. In tumors from *LSL-MYCN;Dbh-iCre* mice a partial gain of murine chromosome 11 was observed, syntenic to human chromosome 17q. Therefore, this model resembles more closely the genetic aberrations observed in human neuroblastomas better than the *Th-MYCN* model which lack any additional chromosomal aberrations.

Since neural crest cells are the suspected embryonic precursor cell in neuroblastoma, Olsen et al., generated neuroblastoma tumors through forced expression of Mycn in neural crest cells. The tumors were phenotypically and molecularly similar to human *MYCN-amplified* neuroblastoma. The neural crest derived neuroblastomas acquired copy number gains and losses that are similar to those observed in human *MYCN*-amplified neuroblastoma. These copy number gains and losses included 2p gain, 17q gain and loss of 1p36. To form tumors in these experiments, they used p53 compromised neural crest cells from the neural tube demonstrating high expression sox10, p75, scl1, and low expression of Th and Phox2b. The embryonic cells were transduced with *MYCN-IRES-GFP* retrovirus and inoculated subcutaneously in mice. With 100% tumor penetrance this proved to be a good, reliable and more rapid method of making a neuroblastoma mouse model (47).

In another study, Alam et al. examined which cell types drive neuroblastoma growth in the *Th-MYCN* transgenic mice model. They showed that both primary tumors and hyperplasia are comprised predominantly of highly proliferative Phox2B^+^ neuronal progenitors. N-myc stimulates the growth of these progenitors by both promoting their proliferation and preventing their differentiation. They also identified a small population of undifferentiated Nestin^+^ cells in both primary tumors and hyperplastic lesions. These cells may serve as precursors of phox2b^+^ neuronal progenitors. Sympathetic neural crest cells express the pro neural genes *Mash1* and P*hox2B*. Mash1 and Phox2b promote further neuronal differentiation by upregulating, the levels of Hand2, Phox2A, and Gata3. These transcription factors collaborate by inducing the expression of Th and dopamine β-hydroxylase, enzymes essential in the catecholamine biosynthesis. Phox2B^+^ in hyperplastic cells from *Th-MYCN* sympathetic ganglia exhibited the morphology of undifferentiated, small round cells and expressed no measurable levels of Th. Alam et al. suggests that Phox2b^+^ hyperplastic cells are halted at the progenitor stage and that Phox2b^+^ neuronal progenitors are the main cellular target of N-myc in driving neuroblastoma expansion from hyperplasia to tumors. Most of the tumor cells expressed Phox2b, and the majority of the Phox2b^+^ tumor cells expressed Ki67, but no measurable levels of Th (58).

Taken together N-myc not only blocks neural progenitors’ differentiation but also promotes the proliferation of Phox2B^+^ neuronal progenitors. This leads to marked increases of the progenitor population in sympathetic ganglia and subsequently the formation of hyperplastic lesions. Alam et al. also proposes that nestin^+^ cells in sympathetic ganglia are possibly the cells of origin for neuroblastoma in *Th-MYCN* mice (58).

The zebrafish model showed that *MYCN* induced neuroblastoma does not develop from the earliest cells populating the superior cervical ganglia. Instead, tumors arise from neuroblasts that migrate into the inter-renal gland later in development, after the kidney has developed. The neuroblasts overexpressing N-myc fail to differentiate, resulting in reduced numbers of chromaffin cells (30).

Neuroblastoma tumors from *Th-MYCN* mice are composed of several cell populations, including Phox2b^+^Th^-^, Phox2b^-^Th^+^, and Phox2b^+^Th^+^ cells. The varying degrees of differentiation in these tumors indicate that the tumors are heterogeneous and may show a molecular resemblance to embryonic stem cells. Sphere forming neuroblastomas were mainly composed of Mycn^+^ and phox2b^+^ cells (114).

## Perspectives and Conclusions

Here, we have emphasized the role of MYCN as an oncogenic driver in neuroblastoma. This is seen in part through MYCN’s ability to initiate stem-like qualities in neural crest-derived cells. A process that is reminiscent of the artificial induction of pluripotency in somatic cells and depends on MYC protein activity. MYCN is significantly involved in the induction of self-renewal by the blockage of differentiation factors as well as by inducing proliferation. While the strong pro-proliferative signal of artificial MYCN overexpression usually leads to apoptosis, the apoptotic machinery in *MYCN* amplified tumors is reorganized in a unique way that allows them to resist apoptotic signals and maybe even benefit from active p53 signaling.

Increased metabolic flexibility by neglecting the Warburg Effect and retaining mitochondrial respiration, as well as, glutamine independence further contributes to the malignancy of *MYCN* amplified tumors and their often observed therapeutic resistance.

The observation that *MYCN* non-amplified tumors never gain extra copies of the *MYCN* gene during their development supports the assumption that a high expression of MYCN in an already established neuroblastoma overloads connected signaling pathways, such as Wnt or HIF signaling.

The extensive and unique restructuring of cellular mechanisms are further reflected by the incompatibility of *MYCN* amplification with other oncogenic events such as genomic rearrangements affecting the *TERT* locus or mutations within the *ATRX* gene. Both might be explained by the fact that the high MYCN expression already deregulates the affected pathways of telomere lengthening, causing mitochondrial dysfunctions and replicative stress. MYCN’s combined effects on DNA damage are incompatible in neuroblastoma (115, 116).

The accumulated data demonstrates that high MYCN expression can act as a single oncogenic driver in neuroblastoma and the mechanisms for how MYCN induces neoplastic transformation has been thoroughly described. However, we still do not know how and at which stage during neural crest development *MYCN* becomes amplified and which cells are affected by sustained high expression of MYCN. The existing animal models are excellent references for studying the mechanisms of induction in tumorigenesis driven by MYCN. Drawbacks of these animal models can be seen however during normal development in which the N-myc expression is controlled by the Dbh or Th promotor. Both are activated relatively late during the maturation of cells within the neural crest and may therefore not fully reflect the initial neoplastic events in humans. Furthermore, for patients with non-amplified *MYCN* neuroblastoma, high expression of MYCN is not correlated with adverse outcomes, in fact the opposite is observed. Instead, a trend correlating high MYCN expression to improved outcomes was evident in these neuroblastoma patients (103). Recent studies indicate that the initial oncogenic event for the development of neuroblastoma must occur early in the neural crest development and no studies to date have identified the precise cell of origin for neuroblastoma. Additionally, which developmental cues or molecular signals and mechanisms are inducing the somatic amplification of the *MYCN* locus at chromosome 2p24.3? Is the gene amplification of *MYCN* the initial genetic aberration in neuroblastoma? Is *MYCN* amplification guided by other earlier genetic aberrations or is the amplification of the chromosome 2p24.3 locus induced by random genomic insults induced by untidy or downregulated DNA repair mechanisms and/or external signals? These are questions that we should address in order to fully understand the biology of *MYCN* amplified neuroblastoma.

## Author Contributions

Conceptualization: JO, CD, and JJ. Writing: JO, CD, and JJ. General review and correction of the manuscript: AP and JJ. Figure Design: AP. Supervision: JJ. All authors contributed to the article and approved the submitted version.

## Funding

This work was supported with grants from the Swedish Childhood Cancer Foundation, the Swedish Cancer Foundation, the Swedish Foundation for Strategic Research (www.nnbcr.se), Märta and Gunnar V Philipson Foundation and The Cancer Research Foundations of Radiumhemmet.

## Conflict of Interest

The authors declare that the research was conducted in the absence of any commercial or financial relationships that could be construed as a potential conflict of interest.
